# BRMS1 in Gliomas—An Expression Analysis

**DOI:** 10.3390/cancers15112907

**Published:** 2023-05-25

**Authors:** Jonas Feldheim, Almuth F. Kessler, Julia J. Feldheim, Dominik Schmitt, Christoph Oster, Lazaros Lazaridis, Martin Glas, Ralf-Ingo Ernestus, Camelia M. Monoranu, Mario Löhr, Carsten Hagemann

**Affiliations:** 1 Section Experimental Neurosurgery, Department of Neurosurgery, University of Würzburg, Josef-Schneider-Str. 11, 97080 Würzburg, Germany; 2 Division of Clinical Neurooncology, Department of Neurology, University Hospital Essen, University Duisburg-Essen, Hufelandstraße 55, 45131 Essen, Germany; christoph.oster@uk-essen.de (C.O.); martin.glas@uk-essen.de (M.G.); 3 Center for Translational Neuro- and Behavioral Sciences, University Hospital Essen, Hufelandstraße 55, 45131 Essen, Germany; 4 Department of Neurosurgery, University Hospital Essen, Hufelandstraße 55, 45131 Essen, Germany; 5 Department of Nuclear Medicine, University of Düsseldorf, Moorenstraße 5, 40225 Düsseldorf, Germany; 6 Department of Neuropathology, Institute of Pathology, University of Würzburg, Josef-Schneider-Str. 2, 97080 Würzburg, Germany

**Keywords:** glioblastoma, metastasis, suppressor, behavior, mRNA, protein

## Abstract

**Simple Summary:**

Gliomas are the most common primary brain tumors and are associated with significant mortality and morbidity. Less than 5% of patients with glioblastoma, the most common glioma histology, survive longer than five years. Therefore, searching for new biomarkers/molecular targets remains a constant issue in glioma research. One such target may be the metastasis suppressor BRMS1. BRMS1 interacts with critical steps of the metastatic cascade in many cancer entities. However, due to the low incidence of extra-cerebral glioma metastasis, the role of metastasis-associated proteins in gliomas remains poorly investigated. Still, the changes in behavior modulated by BRMS1 across different entities (e.g., affecting invasion, migration, and apoptosis) closely resemble the changes seen in gliomas. Additionally, BRMS1’s interaction partners are commonly dysregulated in gliomas. Therefore, BRMS1 shows potential as a regulator of glioma behavior, and we present the first insights into BRMS1 expression in gliomas as a starting point for further investigations.

**Abstract:**

The metastatic suppressor BRMS1 interacts with critical steps of the metastatic cascade in many cancer entities. As gliomas rarely metastasize, BRMS1 has mainly been neglected in glioma research. However, its interaction partners, such as NFκB, VEGF, or MMPs, are old acquaintances in neurooncology. The steps regulated by BRMS1, such as invasion, migration, and apoptosis, are commonly dysregulated in gliomas. Therefore, BRMS1 shows potential as a regulator of glioma behavior. By bioinformatic analysis, in addition to our cohort of 118 specimens, we determined BRMS1 mRNA and protein expression as well as its correlation with the clinical course in astrocytomas IDH mutant, CNS WHO grade 2/3, and glioblastoma IDH wild-type, CNS WHO grade 4. Interestingly, we found BRMS1 protein expression to be significantly decreased in the aforementioned gliomas, while BRMS1 mRNA appeared to be overexpressed throughout. This dysregulation was independent of patients’ characteristics or survival. The protein and mRNA expression differences cannot be finally explained at this stage. However, they suggest a post-transcriptional dysregulation that has been previously described in other cancer entities. Our analyses present the first data on BRMS1 expression in gliomas that can provide a starting point for further investigations.

## 1. Introduction

Tumors of the central nervous system (CNS) represent a significant challenge for modern medicine. They frequently occur in patients of all age categories [1] and can also take a severe clinical course despite intense therapy. Glioblastoma isocitrate dehydrogenase (IDH) wild-type, CNS World Health Organization (WHO) grade 4 (GBM), is the most common malignant primary CNS tumor [2]. Until today, patients diagnosed with GBM face an unfavorable prognosis, even with the intense standard treatment of surgical resection, radiation, and chemotherapy with temozolomide (TMZ) [3,4,5]. In the last decade, only a few phase III trials showed promising results to improve patients’ survival [6,7]. Therefore, identifying new targets and establishing new therapeutic approaches is a major focus of current GBM research [8,9,10,11].

Further challenges in GBM treatment are the frequent recurrences that increase resistance to therapy, multifocal tumor growth that limits the appropriate therapeutic options, and molecular-biological alterations that lead to a different therapeutic response [4,12,13,14,15].

A frequent and diagnosis-defining molecular alteration in CNS tumors is the mutation of IDH. The recent classification by the WHO takes the IDH mutation status into account [16]. Most IDH-wildtype gliomas are now considered GBM [17,18,19]. In contrast, IDH-mutant gliomas are associated with a comparably good prognosis and mainly arise in younger patients classified as astrocytoma IDH mutant, CNS WHO grades 2, 3, or 4 [20,21].

Tumor metastasis is a typical process in oncological diseases, leading to increased morbidity, therapeutical limitations, and, ultimately, the patient’s death [22,23]. Interestingly, gliomas rarely metastasize outside the CNS [24,25], despite sharing multiple attributes with metastatic cells, e.g., highly invasive growth, tumor cell migration, neovascularization, and adaptation to the microenvironment. 

**Figure 1:**
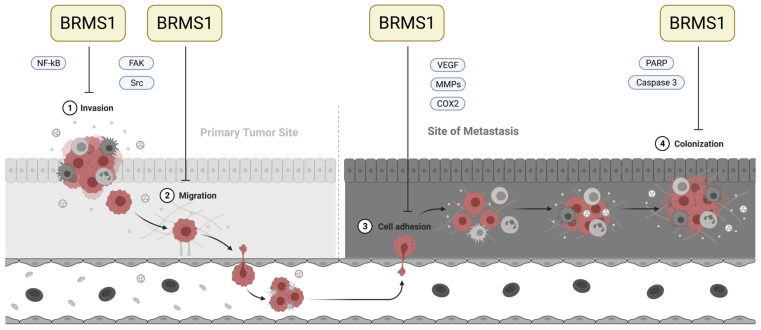
Involvement of BRMS1 in critical steps of the metastatic cascade. Adapted from [40] and created with BioRender.com. BRMS1 interacts with several steps of the metastatic cascade. Its most prominent impact on invasion appears to be through the attenuation of nuclear factor-kappa B (NFκB) function via the classical/canonical pathway [41,42]. One underlying mode of action lies in BRMS1′s ability to bind to the NFκB region of the urokinase plasminogen-activator (uPA) promoter, a downstream target of NFκB, thereby suppressing its (usually) NFκB-dependent gene expression. However, other mechanisms of interaction have also been investigated [42]. Furthermore, BRMS1 expression has been described as inhibiting migratory behavior. In glioma cells, among others, the expression of the Focal Adhesion Kinase (FAK) and Src proteins decreased when BRMS1 was re-expressed [36]. Cell culture experiments further suggest that BRMS1 may interact with VEGF, COX2, and MMPs to prevent cell adhesion and extravasation [42]. After arriving in their target tissue, the disseminated cells need to be able to exit dormancy and begin proliferation again to properly colonize and build a metastasis. BRMS1 can inhibit metastatic outgrowth and has been described as being associated with an increase in PARP and caspase-3 levels, though the molecular mechanisms behind this association have not been exhaustively understood [38,42].

In 2000, Seraj et al. published their discovery of breast cancer metastasis suppressor 1 (BRMS1), encoded on chromosome 11q13 [26]. When repressed in metastatic breast cancer cells, BRMS1 decreased lung and lymph node metastasis in experimental and spontaneous metastasis assays. In contrast, orthotopic tumor growth was only slightly altered, qualifying BRMS1 as a metastasis suppressor [26,27]. Subsequently, these results could be verified and extended to other tumor entities, such as bladder, prostate, rectal, breast, melanoma, ovarian, and non-small cell lung cancer [28,29,30,31,32,33,34,35]. It interferes with multiple steps of the invasion-metastasis cascade, which are also known to be dysregulated in gliomas, namely invasion [29,31,32,36], migration [31,32,36], cell adhesion [28,31,36,37], and colonization at the new site [28,38] (Figure 1).

Additionally, BRMS1 lowers the threshold for tumor cells to undergo apoptosis when exposed to stress [28,38,39]. Due to its multiple effects and interactions, the exact molecular mechanisms triggered by BRMS1 remain the topic of ongoing research.

So far, only a few publications have addressed BRMS1 in gliomas. In 2014, Mei et al. reported BRMS1 protein expression to be significantly decreased in gliomas compared to non-cancerous brain tissue in a tissue microarray [36]. Further, these authors showed that BRMS1 suppressed glioma invasion, migration, and adhesion in cell culture experiments and suggested BRMS1 as a potential future therapeutic target [36]. However, data on BRMS1 in patients’ gliomas is still scarce. To the best of our knowledge, there is no data on BRMS1 mRNA expression or the potential correlation between its expression and tumor and patient characteristics, such as molecular characteristics, growth, relapse pattern, or outcome.

Therefore, we aimed to (1) verify the immunohistochemical results obtained by Mei et al. [36], (2) analyze BRMS1 mRNA expression in gliomas of different WHO grades, and (3) examine the potential correlation of BRMS1 mRNA expression and tumor recurrence, multifocal/unifocal tumor growth, molecular characteristics, outcome, etc. employing our data and bioinformatic analyses of publicly available databases.

## 2. Materials and Methods

### 2.1. Tissue Samples and Clinical Data

We collected tumor and control samples from patients treated in the Department of Neurosurgery, University Hospital Würzburg, Germany. Patients stated their written informed consent for the acquisition of specimens in accordance with the International Conference on Harmonization, the Declaration of Helsinki, as approved by the Institutional Review Board of the University of Würzburg (#103/14). After acquiring the specimens, we froze half of the tissue for mRNA analysis at −80 °C. If a sufficient amount of tissue was provided, we formalin-fixed and paraffin-embedded the other half. The tumor specimens were neuropathologically evaluated, excluded if the tumor cell content was below 80% and classified according to the 2021 WHO classification [16]. We retrospectively collected information on patients’ clinical course, such as sex, age, treatment, tumor localization, relapse or growth pattern, and outcome. Finally, we determined clinical and molecular characteristics associated with patients’ outcomes: We performed semiautomatic tumor volumetry, stained specimens for the proliferation marker Ki67, and examined the methylation of the MGMT promoter by high-resolution melting real-time polymerase chain reaction, as described previously [43,44].

### 2.2. Immunohistochemistry (IHC)

For immunohistochemistry, we cut paraffin-embedded tissue specimens into 3 µm thick slices, dewaxed them twice in xylol (Carl Roth, Karlsruhe, Germany), and rehydrated them in a graded series of ethanol (Carl Roth, Karlsruhe, Germany) diluted in distilled water (100%, 96%, 70%, only distilled water). Afterwards, we boiled the specimens for 10 min at 120 °C in 20 mM citrate buffer (pH = 6.0) (Carl Roth, Karlsruhe, Germany) before treating the slices with 0.7% hydrogen peroxide (Carl Roth, Karlsruhe, Germany) and 10% normal goat serum (Invitrogen, Waltham, MA, USA). Then, we stained BRMS1 using the Envision System HRP DAB (DAKO, Jena, Germany) and the anti-BRMS1 antibody ab65244 (Abcam, Cambridge, UK) in a 1:1500 dilution, according to the manufacturer’s instructions. Finally, we counterstained the cells’ nuclei with hemalaun solution acid according to Mayer (Roth, Karlsruhe, Germany) and embedded the specimens in a xylol-based mounting medium (ORSAtec, Bobingen, Germany).

### 2.3. RNA Extraction and Quantitative Real-Time Polymerase Chain Reaction (qPCR)

mRNA was extracted and converted to cDNA, as described previously [44]. To determine BRMS1 mRNA expression, we performed qPCR on a StepOnePlus Real-time PCR System (Applied Biosystems, Foster City, CA, USA) according to the manufacturer’s instructions. We ran each specimen in triplicates, using a duplex PCR setting that contained TaqMan Universal PCR Master Mix, the internal control GAPDH_VIC_PL (Hs99999905_m1), and the probe BRMS1_FAM (Hs00363036_m1) (all from Applied Biosystems, Waltham, MA, USA), as well as 20 ng of cDNA in each well. The PCR conditions were: 2 min at 50 °C, 10 min at 95 °C, followed by 50 cycles of 15 s at 95 °C and 1 min at 60 °C. We repeated the qPCR if the triplicates exceeded a standard deviation of 0.5 CT.

### 2.4. Software and Statistical Analysis

We prepared the qPCR data with ExpressionSuite Software v.1.0.3 (Thermo Fisher Scientific, Waltham, MA, USA) to unify the qPCR threshold, but used IBM SPSS Statistics 25 (IBM Corporation, Armonk, NY, USA) for all further analyses. We evaluated mRNA expression by the 2^−ΔΔCt^ method based on the mean Ct values of our technical triplicates [45]. While boxplots show the calculated relative expression, the statistical tests were performed on the ΔΔCt—values directly obtained by qPCR. We compared the groups by ANOVA, with Levene’s test to assess the equality of variances and the Dunnet T3 and Scheffe tests as posthoc tests. If the Shapiro-Wilk test revealed that variables were not normally distributed, we evaluated the differences using the Kruskall-Wallis test. We divided the patient collectives by their median BRMS1 mRNA expression and compared both groups’ overall and progression-free survival using Kaplan-Meier analysis (log-rank). We examined the relations between BRMS1 expression and tumor and patient characteristics by ANOVA and non-parametric correlation (Spearman’s Rho). Figure 1 was designed with Biorender (www.biorender.com). The analyses are partly based upon data generated by the TCGA Research Network (https://www.cancer.gov/tcga; accessed on 27 October 2022) exported via the CBioPortal (www.cbioportal.org; accessed on 27 October 2022) and the IVY-GAP database [46]. We retained the subgroups of histologically-distinct anatomic features as defined in the original publication [46]. In addition, we analyzed other publicly available datasets utilizing the GlioVis data portal (http://gliovis.bioinfo.cnio.es/; accessed on 10 May 2023) [47].

## 3. Results

### 3.1. Patient Cohort

We analyzed BRMS1 mRNA expression of 12 non-cancerous brain (NB) specimens (autopsy = 8; epilepsy surgery = 4), four patients with benign adult pilocytic astrocytoma WHO grade 1 (PA), 24 astrocytomas IDH mutant, CNS WHO grades 2 and 3 (in the following referred to as gliomas grade 2/3) and 78 GBM. As explained earlier, we analyzed gliomas grade 2/3 as one combined group, as we could not perform re-classification of these samples according to the most recent WHO guidelines in all cases due to limitations in sample quantity [48]. Samples not initially classified as astrocytoma IDH mutant, CNS WHO grade 2 or 3, were excluded [48]. We retrospectively compiled the clinical data and tumor characteristics of gliomas grade 2/3 (Table 1) and 44 GBM patients (Table 2). The quantity of tumor specimens did not suffice to determine the MGMT promoter methylation for 12 of the latter.

### 3.2. BRMS1 Was Significantly Overexpressed in Gliomas Grade 2/3, Compared to NB, PA and GBM

**Figure 2:**
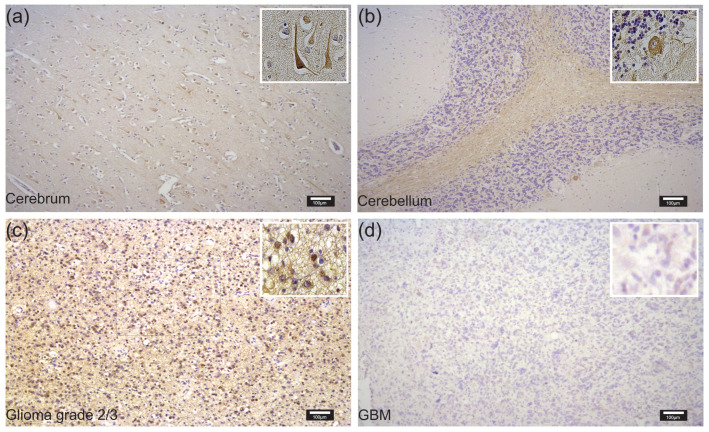
Representative examples of immunohistochemical staining with DAB and a BRMS1 antibody in NB, glioma grade 2/3 and GBM tissue: (**a**) staining of healthy cerebrum; (**b**) healthy cerebellum; (**c**) glioma grade 2/3; and (**d**) GBM. All photographs were taken using the same settings. Magnifications are equal in all four pictures, and scale bars represent 100 µm.

On the protein level, we observed intense BRMS1 staining in the normal cerebrum and cerebellum (Figure 2a,b). which was mainly limited to the neurons of the cortex, the molecular layer, Purkinje cells, and a few glial cells. Staining in the cerebrum appeared slightly stronger than in the cerebellum. Expression in glioma grade 2/3 cells was intermediate (Figure 2c), whereas GBM cells expressed little to no BRMS1 (Figure 2d). We observed clear staining in neurons and glioma cells that was focused on, but not restricted to, the nucleus.

On the mRNA level, the Levene Test revealed an inhomogeneity of variances for all analyses presented in this section (*p* < 0.01), which is why the *p*-values are based on the Dunnet-T3 test that proved a significant difference between the subgroups (*p* = 0.02). NB specimens obtained from autopsies and epilepsy surgery had similar BRMS1 mRNA expression (*p* > 0.05). Therefore, they were combined into one group.

**Figure 3:**
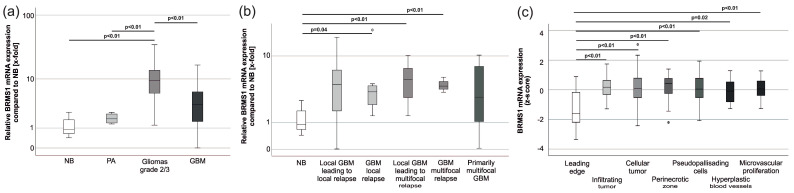
BRMS1 mRNA expression in gliomas. (**a**) BRMS1 mRNA expression in gliomas compared to non-cancerous brain (NB: n = 12; PA: n = 4; gliomas grade 2/3: n = 24, GBM: n = 78; ANOVA: *p* < 0.05, Levene’s test: *p* < 0.05, post-hoc: Dunnet—T3). (**b**) GBM-subgroup analysis of tumors with different growth patterns (NB: n = 12; GBM primary tumor leading to local relapse: n = 24; GBM local relapse: n = 8; GBM primary tumor leading to multifocal relapse: n = 10; GBM multifocal relapse: n = 3; GBM multifocal primary tumor: n = 10; ANOVA: *p* > 0.05). (**c**) IVY-GAP database [46] analysis of BRMS1 mRNA expression in different areas of GBM (leading edge: n = 19; infiltrating tumor: n = 24; cellular tumor: n = 111; perinecrotic zone: n = 26; pseudopalisading cells around necrosis: n = 40; hyperplastic blood vessels in cellular tumor: n = 22; microvascular proliferation: n = 28). Circles represent outliers. Abbreviations: NB, non-cancerous brain; PA, adult pilocytic astrocytoma; GBM, glioblastoma.

BRMS1 mRNA was overexpressed in gliomas grade 2/3 compared to NB (*p* < 0.01, mean 8.9 fold), PA (*p* < 0.01, mean 8.3 fold), and GBM (*p* < 0.01, mean 6.0 fold). Expression in GBM did not significantly differ from expression in NB (*p* > 0.05). Due to BRMS1’s involvement in the metastatic cascade and its role in invasion and migration, we analyzed the GBM panel due to its different growth and relapse patterns. Local relapses (*p* = 0.04, mean 3.5 fold), local tumors leading to multifocal relapses (*p* < 0.01, mean 3.9 fold), and their multifocal relapses (*p* < 0.01, mean 3.1 fold), all displayed overexpression of BRMS1 mRNA, while local tumors leading to local relapses and primarily multifocal tumors had a non-significant tendency towards overexpression of BRMS1 with a broad fluctuation range (Figure 3a,b).

Conclusive with our findings, bioinformatic analyses of the Ivy Gap Database [46] revealed expression differences between different areas of gliomas. BRMS1 mRNA expression was significantly lower in the leading edge than in hyperplastic blood vessels, pseudopalisading cells, areas with microvascular proliferation, cellular tumors, infiltrating tumors, and the perinecrotic zone (Shapiro-Wilk: *p* < 0.01, Kruskal-Wallis: *p* < 0.01, post hoc tests: all *p* < 0.01) (Figure 3c). RNA sequencing and microarray data from other publicly available datasets analyzed via the GlioVis data portal confirmed BRMS1 overexpression in gliomas compared to NB [47,49,50,51,52]. 

### 3.3. BRMS1 mRNA Expression Was Not Associated with Patients’ Survival but Correlated Weakly with Ki67 Staining 

**Figure 4:**
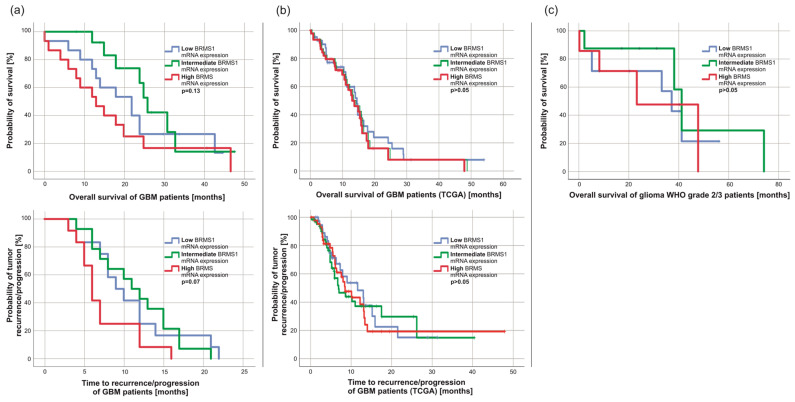
Kaplan-Meier analyses of overall (OS) and progression-free survival (PFS) (**a**) OS (low BRMS1-mRNA expression: n = 15; intermediate BRMS1-mRNA expression: n = 14, high BRMS1-mRNA expression n = 15; Log Rank: *p* > 0.05) and PFS of GBM patients from our collective (low BRMS1-mRNA expression: n = 12; intermediate BRMS1-mRNA expression: n = 14, high BRMS1-mRNA expression n = 12; Log Rank: *p* > 0.05). Six patients died without matching the MRI-based RANO criteria for progress and therefore were excluded from the PFS analyses [53]. (**b**) OS (low BRMS1-mRNA expression: n = 42, intermediate BRMS1-mRNA expression: n = 65, high BRMS1-mRNA expression: n = 45, Log Rank: *p* > 0.05) and PFS of GBM patients from the TCGA dataset (low BRMS1-mRNA expression: n = 42, intermediate BRMS1-mRNA expression: n = 65, high BRMS1-mRNA expression: n = 45, Log Rank: *p* > 0.05). TCGA data were exported from the CBioportal analysis tool [54]. (**c**) OS of glioma grade 2/3 patients (low BRMS1-mRNA expression: n = 7; intermediate BRMS1-mRNA expression: n = 8; high BRMS1-mRNA expression n = 7; Log Rank: *p* > 0.05).

Next, we wondered whether BRMS1 expression had an impact on patients’ survival. However, overall (OS) and progression-free survival (PFS) were similar between GBM patients with high, intermediate, and low BRMS1 expression divided by thirds (Figure 4a, *p* > 0.05). This was confirmed by analyzing the TCGA data (Figure 4b, *p* > 0.05). Similarly, the overall survival of patients with glioma grade 2/3 did not differ between high, intermediate, or low BRMS1 mRNA expression (Figure 4c, *p* > 0.05). We observed a weak statistical correlation between BRMS1 mRNA expression and the percentage of Ki67 positive cells (R = 0.36, *p* = 0.02). Otherwise, BRMS1 appeared to have no association with any of the examined tumor or patient characteristics in our panel (*p* > 0.05), the TCGA dataset, or other publicly available datasets (*p* > 0.05) [46,48,49,50,51]. The BRMS1 mRNA expression between tumors with or without methylation of the MGMT gene promoter was similar (*p* > 0.05).

## 4. Discussion

The term “metastasis” describes the movement of tumor cells from a primary site to colonize distant organs [23]. Metastasis consists of a complex sequence of interrelated steps [55]. To complete this cascade, a metastatic cell must be able to locally invade the surrounding tissue, migrate, form a micrometastasis, and finally colonize its new site. The latter step includes its adaptation to or alteration of the microenvironment and also requires angiogenesis to create a sufficient blood supply [56]. Molecular pathways in this invasion-metastasis cascade may influence metastatic patterns in numerous cancer entities [56]. Although they rarely metastasize, highly invasive growth, tumor cell migration, neovascularization/angiogenesis, and adaptation to the microenvironment are all common and vital characteristics of gliomas [24,25,57,58,59,60,61,62].

Furthermore, glioma cells might migrate long distances in the brain [63] and interconnect in a functional network while growing infiltratively into the surrounding tissue [64]. Therefore, the question arises if metastasis-associated pathways might be promising and so far underestimated targets in understanding and treating gliomas. Few reports describe the influence of metastasis-associated genes and proteins in gliomas [65], yet many attractive targets have not been examined in gliomas in detail.

The metastasis suppressor BRMS1 might be such a target [26,27]. It has been described to interact with common signaling pathways involved in glioma pathogenesis, as focal adhesion kinase, epidermal growth factor receptor, and NFκB [42]. Considering that these pathways target significant steps and functions in the behavior of tumor cells, such as migration, invasion, cell adhesion, or apoptosis [42], it is not surprising that Mei et al. reported decreased BRMS1 protein expression in gliomas and proved that expression of BMRS1 suppresses glioma invasion, migration, and adhesion in cell culture experiments [36]. However, observations on BRMS1 expression, especially at the mRNA level, and regarding potential interrelationships with tumor and patient characteristics are scarce, which prompted us to investigate this topic further. 

We verified that healthy cerebrum and cerebellum specimens stained stronger for BRMS1 than GBM tissue. However, most of this observation was based on BRMS1 expression in neurons, a major component of normal brain tissue yet rare in GBM. As expected, expression in gliomas grade 2/3 was stronger than in GBM, corresponding to their generally less aggressive behavior. Similarly, the tissue of gliomas grade 2/3 includes more neurons and healthy glial cells, which might have contributed to their stronger staining. Interestingly, however, we could also observe multiple BRMS1-positive glioma grade 2/3 cells, whereas tumor cells in GBM and normal astrocytes in the cerebellum/cerebrum rarely displayed strong BRMS1 expression. An interesting future experiment to further determine the expression patterns in different cell types of glioma tissue would be double fluorescence staining with BRMS1 and cell-type-specific markers, such as IDH1R132H, NeuN, GFAP, or CD68.

Surprisingly, these observations did not translate at the mRNA level. Gliomas grade 2/3 had significant BRMS1 overexpression compared to NB, PA, and GBM. Analysis of GBM subgroups revealed that local relapses, local tumors leading to multifocal relapses, and their multifocal relapses also displayed significant BRMS1 overexpression compared to NB when viewed separately. Our bioinformatic analyses support these data. BRMS1 mRNA expression in the leading edge of specimens, potentially the section with the fewest to no tumor cells, was significantly lower than in other areas.

At first glance, these findings on the mRNA and protein levels appear contradictory. However, a similar observation has been made in a cohort of breast cancer patients, where BRMS1 mRNA levels in breast cancer cells were significantly higher than in normal epithelial cells [66] and in hypopharyngeal cancer [67]. Additionally, the expression analyses of healthy brains published by The Human Protein Atlas (www.proteinatlas.org; accessed on 27 October 2022) [68,69,70] show a similar tendency. Curiously, BRMS1 RNA expression in oligodendrocytes, astrocytes, and microglia was reported to be even slightly higher than in excitatory/inhibitory neurons. In contrast, on the protein level, neurons strongly stained for BRMS1, while astrocytes and oligodendrocytes did not (www.proteinatlas.org; accessed on 27 October 2022) [68,69,70]. The solution to this discrepancy may lie in different regulatory mechanisms. BRMS1 expression can be regulated at multiple levels [42]. Apart from transcriptional regulation (e.g., via promoter methylation), BRMS1 expression is influenced at later stages in numerous ways: Micro RNAs, such as miR-423, miR-125a-5p, miR 3200-5p, were found to lower BRMS1 protein levels primarily by binding the 3’UTR region of BRMS1 mRNA [71,72,73,74]. Other mechanisms of BRMS1 regulation include casein kinase 2α, which can trigger degradation of the BRMS1 protein by phosphorylation of serin 30, resulting in cytoplasmatic localization and poly-ubiquitination [42,75]. Therefore, one possible explanation for our findings might be that the lack of BRMS1 is caused by post-transcriptional downregulation of BRMS1 expression, e.g., via protein degradation or micro RNAs. This might lead to compensatory yet insufficient BRMS1 mRNA overexpression that fails to restore a sufficient level of BRMS1 protein. One might speculate that the seeds for this dysregulation are already planted in the healthy brain, as indicated by expression differences between protein/mRNA in oligodendrocytes and astrocytes. However, validation of this hypothesis by follow-up projects is required. 

Survival-time analysis revealed no significant difference between high/low BRMS1 expression groups. One might have expected a different result, as BRMS1 expression has been reported to be correlated with disease-free survival and OS in multiple other tumor entities [32,76,77]. As BRMS1 protein expression in all examined tumor subgroups except for gliomas grade 2/3 appeared to be small to nonexistent, one might argue that expression differences did not matter as they were similarly low. Interestingly, gliomas grade 2/3, the examined malignant tumor type with the comparably best prognosis and most prolonged OS, also displayed the strongest BRMS1 mRNA and protein expression. Therefore, the hypothesis of BRMS1 being associated with glioma patients’ survival should not be completely discarded, though our subgroup analyses indicate otherwise. Publicly available data supported our findings regarding the absence of a correlation of BRMS1 expression with patient and/or tumor characteristics in our and the TCGA datasets. As reported above, we observed a weak statistical correlation between BRMS1 and Ki67 expression that definitely should be noted, yet most likely was the result of a statistical coincidence. Yet, we encourage further examination to investigate whether our interpretation might be accurate. 

## 5. Conclusions

BRMS1 appeared to be dysregulated in gliomas. In concordance with its known mode of action, we could only observe mild to no expression of this metastasis suppressor in GBM. We did not identify any subgroups within the tumor types based on their BRMS1 expression, which indicates that BRMS1 might not be a significant player in these tumors. However, gliomas grade 2/3 displayed surprisingly high expression. BRMS1 protein expression in gliomas appeared to be primarily influenced by post-transcriptional processes. The underlying mechanisms have not been conclusively solved and remain a promising focus of future research.

## Figures and Tables

**Table 1:** Clinical parameters of patients with gliomas grade 2/3 (n = 22).

Sex	Median Age (Quartiles)	Median Overall Survival (Quartiles)
female: 10/45.5% male: 12/54.5%	38.5 years (33.8–48.8 years)	31.0 months (8.0–40.3 months)

Notes: Given are the absolute numbers of patients in each group and the percentage of the analyzed population.

**Table 2:** Clinical parameters of GBM patients (n = 44).

**Patient Characteristics**				
Sex	Female: 19/43.2%		Male: 25/56.8%	
Median age	58.5 years (49.0–69.7 years)			
ECOG	0: 24/54.5%	1: 15/34.1%		>1: 5/11.4%
**Tumor characteristics**				
Median tumor volume	25.5 cm^3^ (15.9–54.3 cm^3^)			
Tumor localization	left hemisphere: 25/56.8%	right hemisphere: 16/36.4%		both hemispheres: 3/6.8%
Tumor localization (lobe)	frontal: 15/34.1%	temporal: 7/15.9%		multiple lobes: 11/25.0%
	occipital: 5/11.4%	parietal: 5/11.4%		cerebellar: 1/2.3%
MGMT promoter methylation	unmethylated: 10/31.3%		methylated: 22/68.8%	
Median Ki67 staining	25% (20–30%)			
**Therapy**				
Time from diagnosis to surgery	0–7 days: 26/59.1%	8–14 days: 10/22.7%		>14 days: 8/18.2%
Surgical intervention	biopsy: 6/13.6%	complete resection: 10/22.7%		incomplete resection: 28/63.6%
Chemotherapy with TMZ	yes: 36/81.8%		no: 8/18.2%	
Radiation therapy	yes: 41/93.2%		no: 3/6.8%	
Treatment in relapse	Best supportive care: 14/36.8%	Systemic treatment (radiation and/or TMZ): 6/15.8%		Surgical resection and systemic treatment: 18/47.4%
**Relapse and outcome results**				
PFS (quartiles)	8.5 months (6.0–13.3 months)			
Relapse	primarily multifocal: 6/13.6%	local relapse: 26/59.1%		multifocal relapse: 12/27.3%
OS (quartiles)	18.0 months (12.0–25.8 months)			

Notes: The absolute numbers of GBM patients in each group and the percentage of the analyzed population are given. The quantity of remaining tumor specimens did not suffice to determine the MGMT promoter methylation for 12 patients. Abbreviations: GBM, glioblastoma; ECOG, Eastern Cooperative Oncology Group scale; TMZ, temozolomide; MGMT, O-6-methylguanine-DNA methyltransferase; PFS, progression-free survival; OS, overall survival.

## Data Availability

All data are contained within the manuscript. Raw data are available upon reasonable request from the corresponding author.
